# Mobile Apps for Older Adults: Systematic Search and Evaluation Within Online Stores

**DOI:** 10.2196/23313

**Published:** 2021-02-19

**Authors:** Alexandra A Portenhauser, Yannik Terhorst, Dana Schultchen, Lasse B Sander, Michael D Denkinger, Michael Stach, Natalie Waldherr, Dhayana Dallmeier, Harald Baumeister, Eva-Maria Messner

**Affiliations:** 1 Department of Clinical Psychology and Psychotherapy Institute of Psychology and Education University of Ulm Ulm Germany; 2 Department of Psychological Research Methods Institute of Psychology and Education University of Ulm Ulm Germany; 3 Department of Clinical and Health Psychology Institute of Psychology and Education University of Ulm Ulm Germany; 4 Department of Rehabilitation Psychology and Psychotherapy Institute of Psychology University of Freiburg Freiburg im Breisgau Germany; 5 Agaplesion Bethesda Clinic Geriatric Research University of Ulm Ulm Germany; 6 Institute of Databases and Information Systems University of Ulm Ulm Germany; 7 Department of Epidemiology Boston University School of Public Health Boston, MA United States

**Keywords:** mHealth, MARS, MARS-G, older adults, mobile apps, apps, aging

## Abstract

**Background:**

Through the increasingly aging population, the health care system is confronted with various challenges such as expanding health care costs. To manage these challenges, mobile apps may represent a cost-effective and low-threshold approach to support older adults.

**Objective:**

This systematic review aimed to evaluate the quality, characteristics, as well as privacy and security measures of mobile apps for older adults in the European commercial app stores.

**Methods:**

In the European Google Play and App Store, a web crawler systematically searched for mobile apps for older adults. The identified mobile apps were evaluated by two independent reviewers using the German version of the Mobile Application Rating Scale. A correlation between the user star rating and overall rating was calculated. An exploratory regression analysis was conducted to determine whether the obligation to pay fees predicted overall quality.

**Results:**

In total, 83 of 1217 identified mobile apps were included in the analysis. Generally, the mobile apps for older adults were of moderate quality (mean 3.22 [SD 0.68]). Four mobile apps (5%) were evidence-based; 49% (41/83) had no security measures. The user star rating correlated significantly positively with the overall rating (*r*=.30, *P*=.01). Obligation to pay fees could not predict overall quality.

**Conclusions:**

There is an extensive quality range within mobile apps for older adults, indicating deficits in terms of information quality, data protection, and security precautions, as well as a lack of evidence-based approaches. Central databases are needed to identify high-quality mobile apps.

## Introduction

Demographic change continues worldwide [1]. Globally, the proportion of older adults, those aged 65 years and older [1,2], will increase more than 60% until the year 2030 [1]. In 2050, it is estimated that 1.6 billion people (16.7% of the total world population) will be age 65 years or older [1]. The global aging population poses a variety of challenges to health care systems and their sustainability, such as increasing costs and potential medical and social undersupply to older adults due to a lack of health care professionals in the future [3,4]. Moreover, older adults are confronted with challenges such as physical and cognitive functional impairments, changes in social relationships, socioeconomic status, and loneliness [5]. These age-related changes often have far-reaching effects on overall health, preservation of independence, and ability to participate socially [5]. Some older adults might need assistance in retaining an active and independent lifestyle, sustaining physical and mental performance, preventing physical and mental disorders, and maintaining an appropriate system of social support [3,6].

Mobile and internet technologies such as mobile apps offer possible approaches to increase the empowerment of older adults, support social activities, prevent cognitive and physical decline, decrease loneliness, and provide assistance in everyday activities [7-12]. Mobile apps could be innovative solutions to help older adults maintain independence and enable them to promote their health and functioning [8,9,13,14].

Mobile apps may offer many advantages for older adults to complement traditional health care behavior, as they can be cost-effective if implemented on a large scale and used independently of time and location [15,16]. Furthermore, they have the potential to simplify social and medical care, which could contribute to the promotion of social inclusion and support living at home on a longer term [8-10,17].

Nevertheless, uptake and acceptance of mobile apps by older adults are rather low [18]. This may stem from various risks of mobile app use and barriers to uptake, including concerns about the quality and benefits of mobile technologies, accuracy of provided information, fear of misdiagnosis, worries about data misuse and insecurity regarding data transmission, costs of use, qualification of the app developers, lack of evidence, and poor usability [18-23]. Also, older adults occasionally show a lack of perceived self-efficacy regarding mobile app use, which negatively influences uptake [16,18].

Smartphones have become an integral part of everyday life, even for older adults [24,25]. In 2017, 40% of Americans aged 65 years and older were using a smartphone [24]. Two years later, in 2019, 73% of Germans aged 60 to 69 years used a smartphone [26]. Many studies imply that due to the aging of the baby boomer generation, more older adults will use smartphones [18,27]. As a result, mobile apps could reach a large number of older adults in the future [18,27].

There are many mobile apps available in the app stores [28], but the quality of publicly available mobile apps for older adults has not been systematically evaluated so far. There is only one systematic review that reports the quality of publicly available mobile apps for the promotion of balance in older adults, which concluded that mobile apps are of acceptable quality [29]. However, this review has a narrow scope as it only focused on improving balance in older adults through mobile apps, and there are presently no further systematic reviews of mobile apps for older adults available. Therefore, information about the quality, content, and data handling in mobile apps for older adults is not available to date.

Users can have problems identifying mobile apps that will effectively and safely support them in their health care [30]. This is mainly caused by the vast number of available mobile apps, opaque dynamics in the app stores, and the perceived lack of technical knowledge in older adults [30,31]. User star ratings from the app stores seem to be a questionable indicator for quality as they can originate from fictional persons and seem to be mostly determined by functionality and aesthetics [32,33].

To close this research gap, our study has systematically searched for mobile apps in the European app stores with a focus on older adults. Hence, their general characteristics, aims, methods, content, and quality were assessed using a multidimensional instrument, the German version of the Mobile Application Rating Scale (MARS-G) [34,35]. To evaluate various acceptance barriers that discourage older adults from using a mobile app, this systematic review focuses on the following characteristics of mobile apps for older adults in the European commercial app stores:

- Privacy and security features
- Quality criteria based on the MARS-G (engagement, functionality, aesthetics, information)
- Correlation between the user star rating and the MARS-G overall rating
- Prediction of overall quality due to the obligation to pay fees

## Methods

### Study Design

The systematic review was based on the Preferred Reporting Items for Systematic Reviews and Meta-analyses (PRISMA statement) according to Moher and colleagues [36], with discrepancies due to the characteristics of mobile apps (for details see Multimedia Appendix 1).

### Search Strategy and Inclusion Criteria

A web crawler was used to systematically screen the European Google Play and App Store for eligible mobile apps with the search terms “old,” “dementia,” “memory,” “mnemonic,” “elderly,” “senior,” “maturity,” “retiree,” “seniority,” and “aided recall.” The search string to identify mobile apps for older adults resulted from findings of self-conducted focus groups with older adults, caretakers, and physicians followed by an expert discussion (EMM, LS, HB, MD, DD, and NW). The web crawler is a search engine that systematically searches the internet and country-specific app stores such as Google Play and the App Store for eligible mobile apps [37]. The search was conducted on February 5, 2019.

All identified mobile apps were listed in a central database, and the first results were screened by the reviewers (AP, DS, MD, MS, LS, DD, and NW). The screening was conducted via an Access (Microsoft Corp) file. Every mobile app was screened by two reviewers. Disputes were discussed with a supervisor (EMM). To be included in this review, mobile apps had to meet the following inclusion criteria: (1) designed for older adults or older adults, their caregivers, and relatives; (2) available and downloadable in the official Google Play or the App Store; (3) in German or English (in accordance with the reviewers’ language skills); (4) functional to enable an assessment (eg, no device problems); and (5) usable independently of other software (eg, software on smartwatches). Duplicates were automatically and manually excluded. Nonworking links were tried several times. The reviewers excluded mobile apps that did not meet the inclusion criteria according to the title, mobile app description, given images, or comments of mobile app users in the app stores in the first step.

On May 8 and 9, 2019, an additional manual search of mobile app recommendations in the app stores took place by a reviewer (AP) to identify further relevant mobile apps. This should ensure an up-to-date and comprehensive search for mobile apps. Additionally to the previous search terms, the following German and English search terms were used: “seniors,” “older adults,” “Alzheimers,” “memory games,” “retirement,” “pills,” “dementia,” “memory,” “senior health,” and “emergency call.” The search terms to identify mobile apps for older adults resulted from findings of self-conducted focus groups and were developed in an expert discussion (EMM, LS, HB, MD, DD, and NW). In addition to technical terms, relevant synonyms and alternatives used by end users were added to the extracted search terms [38]. These mobile apps were also reviewed for their entitlement to be included in the analysis.

For the MARS-G analysis, the mobile apps were downloaded and checked regarding the inclusion criteria and their functionality for the review (eg, no device problems). Technical problems were validated on at least two devices. The mobile apps were downloaded and installed either on an iPad mini (Apple Corp; model MK9N2FD/A; operating system 12.1), a MediaPad X2 (Huawei Device Co; model GEM-701L; operating system 5.0.1), or an iPhone 6 (Apple Corp; model A1586; operating system 12.2).

### Data Collection Process

The quality assessment of the mobile apps was conducted by two independent reviewers (AP, DS, MD, MS, LS, DD, or NW) using the MARS-G [35]. Prior to the rating, the reviewers received standardized online training, which is publicly accessible and free of charge [39]. Each mobile app had been explored and used for at least 15 to 20 minutes to examine the functionality, content, and quality. The quality rating took about 30 minutes for each mobile app and was documented via an Access file. Reviews were completed on May 28, 2019. For quality assurance, interrater reliability was calculated. Rater agreement was examined by intraclass correlation (ICC) based on a 2-way mixed-effect model with absolute agreement. When the ICC was below a minimum value of .75 [40] or when there were disputes between the reviewers, a third reviewer was consulted [34,35].

### Evaluation Tool

The MARS-G evaluation tool is a reliable and valid scale for the quality assessment of mobile apps [35,41]. The MARS-G shows a good to very good internal consistency for all subdimensions (ω=.72-.90) as well as for the overall score (ω=.82, 95% CI .76-.86) and a high ICC (2-way mixed ICC .84, 95% CI .82-.85) [35]. The correlations of the corresponding dimensions of the MARS and MARS-G range from *r*=.92-.98 [35].

### General Characteristics

The classification page of the MARS-G was used to examine mobile app characteristics. It contains descriptive and technical information about the mobile app: (1) name, (2) platform, (3) content-related subcategory, (4) store link, (5) price, (6) user star rating, (7) aims, and (8) methods [34,35].

### Data Protection and Security Precautions

The assessment of privacy and security features based on MARS-G is on a descriptive level (eg, availability of privacy policy, imprint). All features were assessed based on downloaded mobile apps, and only information that was disclosed within the mobile app or its description in the app stores was investigated.

### Categorization

The categorization of mobile apps for older adults according to Cunha and colleagues [42] was used for the analysis to enable a classification independent of the app stores. This classification was developed using a methodological search in Google Play and the App Store for mobile apps designed to help older adults [42]. Table 1 lists the various categories with examples of content topics.

**Table 1:** Mobile app categories for older adults with exemplary topics according to Cunha et al [42].

Categories	Exemplary topics
Diagnostic	Cognitive impairments, physical and mental illnesses
History	Monitoring of vital parameters such as blood pressure, and organization of daily activities
Improve	Relaxation, speech-to-text, text-to-speech, risk assessment, magnifying glass, medication recognition, pictogram-to-speech, communication portals, and social networks
Informative	Healthy living, education, and psychoeducation about mental and physical illnesses
Interface	Mobile apps for conversion to a user-friendly interface
Measurement	Physical activity, pedometer, and GPS tracking
Protection	Drug reminder, help requests, and localization
Simulation	Simulation of diseases, impairments, or appearance
Trainer	Memory, relaxation, logical thinking, fitness, and cognitive speed
Tutorial	Accident rehabilitation, sign language, improvement of self-esteem, and improvement of communication

### Quality Assessment

The multidimensional quality rating of the MARS-G includes 19 items on 4 different subdimensions, which are evaluated on a 5-point Likert scale (1=inadequate, 2=poor, 3=acceptable, 4=good, and 5=excellent): (1) engagement—5 items (entertainment, interest, individual adaptability, interactivity, target group); (2) functionality—4 items (performance, usability, navigation, motor and gestural design); (3) aesthetics—3 items (layout, graphics, visual appeal); and (4) information—7 items (accuracy of app description, goals, quality of information, quantity of information, quality of visual information, credibility, evidence base) [34,35].

### Data Analyses

For the evaluation of the overall rating and quality, the total score was calculated from the 4 subdimensions [34]. The ratings of the reviewers were averaged for all calculations. Mean scores and standard deviations were calculated for the MARS-G overall rating and subdimensions.

Item 19 on the information subdimension was used to assess whether empirical studies were available for a mobile app. This item was investigated by searching the mobile app name in Google Scholar, PubMed, Google, and the developers’ or providers’ websites for existing efficacy and effectiveness studies [34].

Bivariate correlations between the user star rating and the MARS-G ratings were calculated. Also, bivariate correlations between the user star rating and the number of security and privacy measures were determined. The user star ratings were extracted from the app stores. The user star rating from Google Play and the App Store can be assigned on a scale of 1 to 5 stars and is displayed to mobile app seekers in the app stores as a cumulative average of individual ratings [43]. Mean score and standard deviation were calculated for the user star rating.

To examine whether the obligation to pay fees is a predictor of overall quality, an exploratory regression analysis was conducted in which the predictor was dummy coded (1=obligation to pay fees, 0=no obligation to pay fees). Mobile apps that required an initial payment for use were defined as “obligation to pay fees.” Mobile apps that were not priced at the time of purchase or had a free basic version were defined as “no obligation to pay fees” [44,45].

A *t* test for independent samples was used to check whether the mobile apps from the app stores differ regarding their MARS-G overall and subdimension mean value. For all analyses, an alpha level of 5% was defined [46]. All statistical analyses were performed using SPSS Statistics 24 (IBM Corp) and R (R Foundation for Statistical Computing).

## Results

### Search

**Figure 1:**
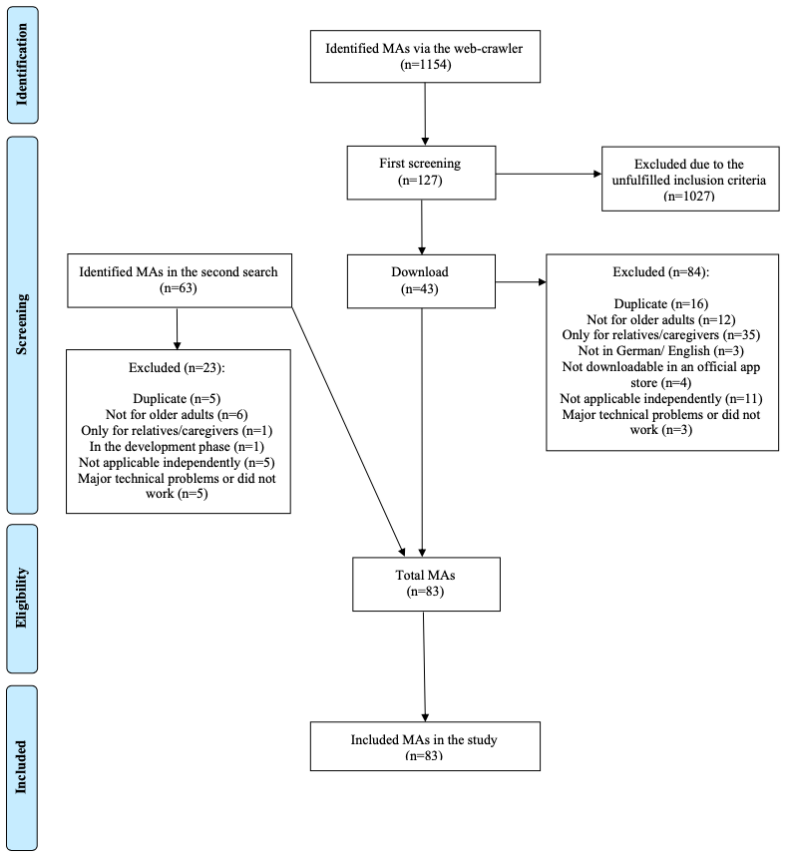
Flowchart of the mobile app selection process.

The web crawler identified 1154 mobile apps, of which 11.01% (127/1154) were found to be eligible by initial screening (Figure 1). Due to the unfulfilled inclusion criteria, 88.9% (1027/1154) of mobile apps were excluded. After the initial screening, 127 mobile apps were downloaded, of which 66.1% (84/127) did not meet the inclusion criteria (eg, duplicates, only for relatives and caregivers), leaving 33.9% (43/127) to be included in the MARS-G analysis. In an additional manual search, 63 mobile apps were detected, of which 37% (23/63) were excluded. In summary, 6.82% (83/1217) of mobile apps found were included in the analyses (for details on the included mobile apps see Multimedia Appendix 2).

### General Characteristics

Of the mobile apps, 64% (53/83) were from Google Play and 36% (30/83) were from the App Store. There were no significant mean differences in the MARS-G overall rating between mobile apps from different stores (t_81_=1.399, *P*=.17). Furthermore, there were no significant mean differences in the individual subdimensions of the MARS-G rating for mobile apps from different app stores (for all calculations *P*>.05). Most of the mobile apps were free of charge (73/83, 88%); 12% (10/83) were priced. The average price was €0.75 (SD 2.76), ranging from €0 to €18.99 (US $0 to $23.32). The 69 existing user ratings from the app stores had an average score of 4.15 (SD 0.70). Of the mobile apps, 37% (31/83) were designed for prevention, 41% (34/83) for treatment, 31% (26/83) for rehabilitation, 27% (22/83) for aftercare, and 60% (50/83) for assistance in everyday life. Multiple naming of fields of application for one mobile app was possible. A total of 31% (26/83) were developed and published by a legitimate source (such as a nonprofit organization or university). None of the mobile apps were developed with the help of competitive government or research funding.

**Figure 2:**
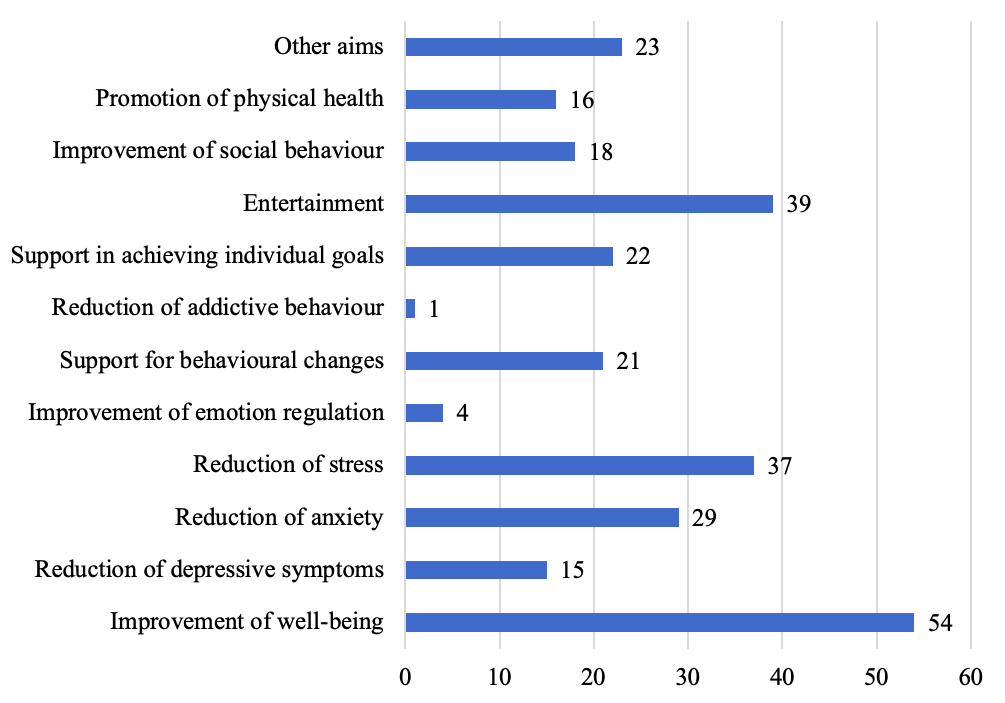
Frequency of objectives of mobile apps for older adults. Multiple naming of objectives for one mobile app was possible. Data are given for n=83 mobile apps.

On average, the mobile apps for older adults had 3.36 (SD 1.79) aims, with a maximum of one mobile app having 8 aims. Most common aims were improvement of well-being (54/83, 65%), entertainment (39/83, 47%), reduction of stress (37/83, 45%), and reduction of anxiety (29/83, 35%). Aims classified under other aims (23/83, 28%) included, for example, disease education (2/83, 2%) and screening for Alzheimer disease (3/83, 4%). Figure 2 provides an overview of the frequency of aims in mobile apps for older adults.

**Figure 3:**
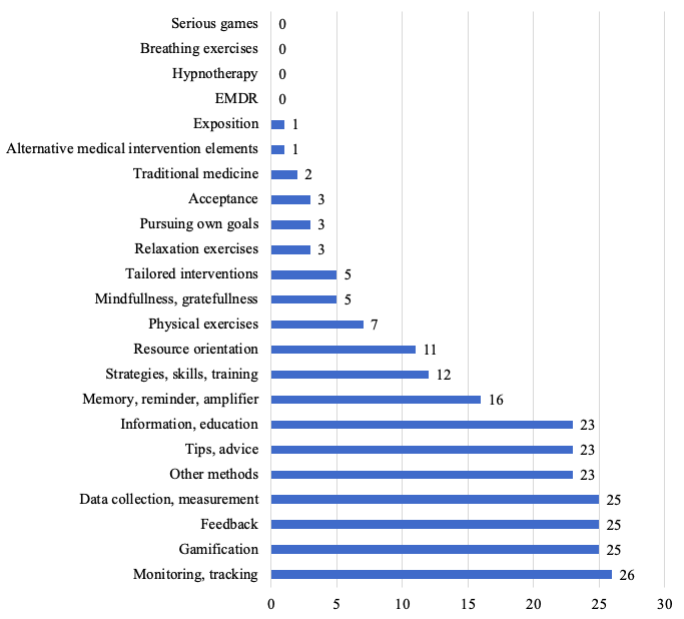
Frequency of methods used in mobile apps for older adults. Multiple naming of different methods in one mobile app was possible. Data are given for n=83 mobile apps.

On average, the mobile apps used 2.88 (SD 1.81) methods. The number varied from 1 to 9 methods. The most common methods were monitoring and tracking (26/83, 31%), data collection and measurement, feedback, and gamification (each 25/83, 30%) as well as information and education and tips and advice (each 23/83, 28%). Some mobile apps included memory, reminder, amplifier (16/83, 19%), strategies, skills, training (12/83, 14%) and resource orientation (11/83, 13%). Only a few mobile apps included physical exercises (7/83, 8%), mindfulness and gratefulness, and tailored interventions (each 5/83, 6%), acceptance, pursuing own goals and relaxation exercises (each 3/83, 4%), and traditional medicine (2/83, 2%) or alternative medical intervention elements and exposition (each 1/83, 1%). Methods classified under other methods (23/83, 28%) included, for example, personalization (7/83, 8%), social networking features (4/83, 5%), and emergency button and contacts (1/83, 1%). None of the mobile apps included serious games, breathing exercises, hypnotherapy or EMDR. Figure 3 illustrates the frequencies of used methods in mobile apps for older adults.

### Data Protection and Security Precautions

The average number of security and privacy measures was 2.07 (SD 2.76). Of the included mobile apps, 49% (41/83) had no data protection precautions. Most frequently (30/83, 36%), a contact, contact person, or imprint was given. Only in 7% (6/83) emergency functions were available; 5% (4/83) provided data transmission security. Table 2 provides an overview of all data protection precautions in the mobile apps.

**Table 2:** Privacy and security measures found in mobile apps.

Data protection precaution	Value^a^, n (%)
Allows password use	22 (27)
Requires a log-in	20 (24)
Has a privacy statement	28 (34)
Requires active confirmation of a consent form	14 (17)
Information on dealing with the data	14 (17)
Notes on financing/conflict of interest	14 (17)
Contact/contact person/imprint	30 (36)
Data transmission security	4 (5)
Emergency functions available	6 (7)
Security strategies for mobile phone loss	20 (24)
Other security strategies	0 (0)

^a^Multiple naming of different data protection precautions for one mobile app are possible.

### Categorization

According to the categorization of Cunha and colleagues [42], a majority (31/83, 37%) of the mobile apps could be classified as trainer. Overall, 16% (13/83) were classified as protection, 11% (9/83) as interface, 10% (8/83) as informative, and 7% (6/83) as improve. Only a few mobile apps were found in the categories measurement (2/83, 2%), history (4/83, 5%), and diagnostic and tutorial (each 5/83, 6%). None of the mobile apps could be classified as simulation. The best overall quality was found for the categories measurement (mean 3.77 [SD 0.15]), diagnostic (mean 3.67 [SD 0.75]), and trainer (mean 3.28 [SD 0.82]). However, overall quality for categories informative (mean 3.24 [SD 0.29]), tutorial (mean 3.23 [SD 0.45]), protection (mean 3.18 [SD 0.59]), improve (mean 3.13 [SD 0.59]), interface (mean 2.86 [SD 0.44]), and history (mean 2.82 [SD 0.97]) was poor to moderate.

### Quality Assessment

**Figure 4:**
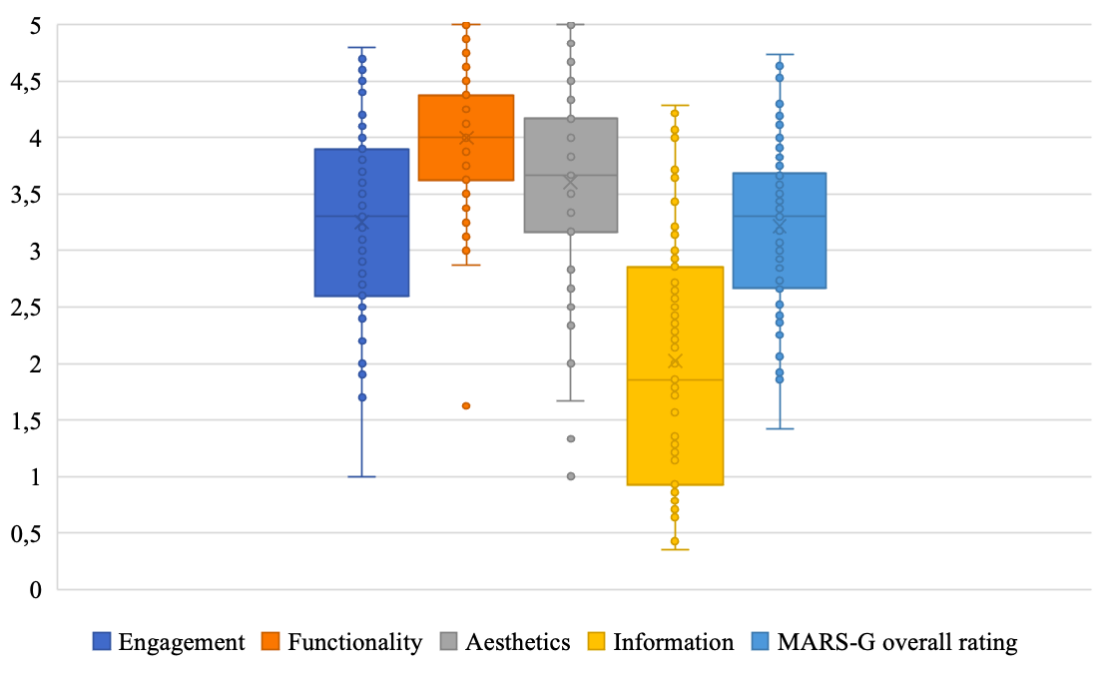
Graphical representation of the distribution of the Mobile Application Rating Scale (German version) overall rating, and the four subdimensions. The median, the interquartile distance as well as the range and outliners were given (n=83 mobile apps).

The overall rating showed an excellent level of interrater reliability (2-way mixed ICC .97, 95% CI .97-.98). According to Portney and Watkins [47], the interrater reliabilities of the MARS-G subdimensions were excellent (ICC .91-.99). The overall quality of the mobile apps for older adults was moderate, with a mean quality of 3.22 (SD 0.68). The subscale engagement was moderate (mean 3.25 [SD 0.82]), functionality good (mean 3.99 [SD 0.59]), aesthetics moderate to good (mean 3.60 [SD 0.85]), and information quality poor (mean 2.02 [SD 1.10]). Figure 4 shows a graphical representation of the distribution of ratings for overall quality and individual subdimensions.

Significant positive bivariate correlations were found between overall rating and subdimensions (*r*=.68–.85, *P*<.001). A correlation table is presented in Table 3.

**Table 3:** Correlations between the mean values of the four MARS-G subdimensions, overall rating and user star rating.

Characteristics		MARS-G^a^									
		Engagement	*P* value	Functionality	*P* value	Aesthetics	*P* value	Information	*P* value	Overall rating	*P* value
**MARS-G**											
	Engagement	—^b^	—	—	—	—	—	—	—	—	—
	Functionality	.52	<.001	—	—	—	—	—	—	—	—
	Aesthetics	.62	<.001	.54	<.001	—	—	—	—	—	—
	Information	.55	<.001	.33	.002	.58	<.001	—	—	—	—
	Overall rating	.83	<.001	.68	<.001	.85	<.001	.83	<.001	—	—
User star rating^c^		.27	.03	.11	.38	.19	.13	.32	.01	.30	.01

^a^MARS-G: German version of the Mobile Application Rating Scale.

^b^Not applicable.

^c^Correlations were calculated with 69 mobile apps since the user star rating was missing for 14 apps.

### Quality Rating on Evidence

Four (5%) mobile apps were evidence-based. For Lumosity [48,49] and NeuroNation [50], various efficacy studies, mainly for the web-based versions, in the form of randomized controlled trials with different participant groups (eg, age, health status, ethnicity) exist. These studies suggest significant improvements in different cognitive performances as processing speed or short-term memory due to training with these mobile apps. However, only a few studies met the minimal standards of a randomized controlled trial (eg, random assignment of participants) [51]. For MindMate and Constant Therapy, a significantly positive difference in therapeutic success could be shown compared with conventional or no training in older adults with cognitive impairments [52,53].

### Association Between User Star Rating and Quality of Mobile Apps

The user star rating and overall rating correlated significantly positively with *r*=.30 (*P*=.01). Furthermore, there was a significant positive relationship between the user star rating and the subdimensions engagement (*r*=.27, *P*=.03) and information (*r*=.32, *P*=.01). The user star rating did not correlate significantly with the number of security and privacy measures (*r*=.09, *P*=.49). The correlations were calculated with n=69 mobile apps since the user star rating was missing for 14 apps.

### Exploratory Regression Analysis

There were no bivariate correlations between the overall rating or the four subdimensions and the obligation to pay fees (*P*>.05). The obligation to pay fees had no predictive value for overall quality (β=.07, *F*_1,81_=0.098, *P*=.75, adjusted *R*^2^=.01%).

## Discussion

### Principal Findings

In this study, we systematically examined the quality of 83 mobile apps for older adults in the European commercial app stores using a reliable and valid rating instrument. Furthermore, we assessed general characteristics, aims, methods, content, and privacy and security measures of the mobile apps for older adults. In general, the mobile apps were of moderate quality with a wide range of quality ratings. This result is in line with findings from other systematic mobile app reviews using the MARS [29,54-56]. The pattern of high functionality and low information quality of the mobile apps for older adults is in accordance with other MARS studies [55,57]. However, previous research on mobile apps for older adults implies a low functionality of these [17]. This result might point out the improvement of mobile app functionality over the past years.

The generally low information quality with a wide range is also in line with the results of other systematic reviews [38,55]. The included mobile apps often did not refer to the authors or sources of information, and the actuality and correctness of the information were not guaranteed. The decreased information quality is associated with various risks for mobile app users, mainly because misinformation can result in incorrect self-diagnosis and adverse health decisions in prevention, health promotion, and treatment [58,59].

Moreover, users are confronted with data and security issues, as 49% of the mobile apps contained no security or data protection measures, and those that do exist lack clarity. The literature implies that concerns about the lack of data protection measures represent an essential usage barrier for older adults [18,21]. Sunyaev and colleagues [60] suggested that mobile apps used in health care systems contain highly sensitive data and should, therefore, be subject to particularly strict data protection guidelines. In their assessment of mobile apps that provide health advice, they found that only 30.5% of mobile apps had privacy policies, of which two-thirds did not specifically address the content of the mobile apps, but commercial rights, distribution rights, or third-party rights [60]. This indicates a lack of transparent reporting on how mobile apps handle personal and health-related data. Therefore, the risk that the data can be evaluated, merged with other data, or passed on to third parties without the mobile app users’ knowledge is given [61,62]. Even if mobile apps had a privacy policy, many mobile apps transmitted data services provided by Facebook or Google [63]. In particular, mobile apps that offer interface and protection should guarantee the privacy and security of data transmission. However, compliance with these guidelines is currently not ensured.

Furthermore, the efficacy and effectiveness of mobile apps for older adults are poorly examined [64]. Only 5% of mobile apps had evidence for their efficacy [48-50]. This small number is in line with the results of some systematic health-related mobile app reviews [38,56,65]. The limited emergence of evidence-based mobile apps can partly be explained by the fact that the evaluation methods for health interventions, such as randomized controlled trials, are time-consuming and cost-intensive [66,67]. Also, most mobile apps in this study, as well as mobile apps for other target groups, came from private sector companies without scientific background on the specific context [55,68-70]. Many mobile apps developed by universities and research projects do not enter the mobile app market or are not included in the top rankings due to lower download rates [68,71]. Interdisciplinary cooperation between health care providers, health insurance companies, and researchers would be essential to reach older adults in need who might benefit from a high-quality mobile app.

Top-ranked mobile apps often have a high user star rating, which is discussed as an indicator of mobile app quality [72]. This study found a moderate positive correlation between user star rating and overall rating as well as the subdimensions engagement and information, which is in accordance with some systematic reviews [73] but not with others [32,57]. These results indicate that engagement and information quality might play an essential role in the rating of mobile apps by older adults. The facets of the MARS subdimension engagement, such as entertainment, individual adaptability, interactivity, and target group specificity, are cited as essential principles for the development of mobile apps for older adults and have been associated with the effectiveness of health interventions in several studies [64,74-77]. In previous studies, users were described selecting mobile apps according to the quality of the aesthetics and functionality, which could not be replicated in this study [33,78]. Mobile apps for older adults might be thoroughly checked regarding their content and quality before older adults use them. However, there was no correlation between the user star rating and the number of data security measures, which suggests that the user star rating is not an indicator of data protection and privacy and vice versa. Furthermore, user star ratings could originate from fictitious persons, and each person could apply a different focus of evaluation (eg appearance, usability) [79]. Besides, user star ratings from app stores could refer to previous versions of a mobile app, which does not guarantee that the mobile app is up to date and may cause distortions due to evaluations of different versions [43]. Therefore, the user star rating does only seem to be a limited orientation aid for the selection of a mobile app. Other strategies for selecting a mobile app should be considered.

According to our results, the obligation to pay fees did not predict mobile app quality. In previous studies, it was partly implied that paid mobile apps are more credible, trustworthy, and recommendable and are more likely to promote users’ health and well-being [57,80]. Other studies could also not find an association between the obligation to pay fees and mobile app quality [32,69]. Since the cost of mobile app use represents an important barrier for the uptake of mobile technologies as mobile apps by older adults [18,21], it is beneficial that there are no significant differences in quality.

Most mobile apps could be assigned to the trainer category. Training mobile apps such as fitness and cognitive exercises for the prevention of neurodegenerative diseases as well as social media mobile apps are mostly used by older adults [18,81]. In previous studies, mobile health interventions for older adults containing preventive training and mechanisms for behavioral changes, self-management of chronic diseases, and social inclusion have had a positive effect on self-confidence, health, performance, and general well-being of older adults [10,76,82-85]. In this study, most of the mobile apps were designed to support the daily lives of older adults (eg, entertainment and family connectivity) as well as for rehabilitation and treatment of diseases (eg, symptom tracking and medication). Thereby, most of the mobile apps focused on methods such as monitoring and tracking, feedback, data collection and measurement, information and education, or gamification. Various studies implied the importance of these methods for the effectiveness of mobile apps, use behavior and adherence, interaction, and motivation in the use of mobile apps by older adults [10,68,76-78, 86-88].

### Strengths and Limitations

One strength of this study is the use of traditional systematic review methodology, such as systematic search, independent screening, and quality evaluation of the included mobile apps on a reliable scale. The multidimensional MARS-G enabled an objective, reliable, and valid rating [35,41]. The categorization, according to Cunha et al [42], made it possible to classify the mobile apps specifically for older adults independently of the app stores. Also, the additional manual exploration of mobile apps in the app stores ensured an up-to-date and comprehensive search. In this way, a realistic search for mobile apps by older adults and their relatives could be simulated. The use of nonprofessional and technical terms made it possible to cover a wide range of mobile apps in the search terms.

However, due to the high frequency of new and further developments as well as the continuous technological progress of the mobile app market [58], this study shows a current snapshot of the quality of mobile apps for older adults. Some of the included mobile apps may no longer be downloadable, their content may have changed, new versions could be available, or new mobile apps may have been developed during the publication of this study, therefore reducing the actuality of this rating.

Another limitation is the country-specific search for mobile apps in the German and British app stores. Different mobile apps are offered in various countries since the selection of countries in which a mobile app is available is determined by the developers [89]. This could limit the generalizability of the results of our study [90].

Furthermore, mobile apps were not tested for a longer time, as in days or weeks. Therefore, some aspects of the mobile apps may not have been detected, and some errors may have remained hidden.

Additionally, we assessed privacy and security measures on a descriptive level, and the included data is based on information within the mobile apps and description in the app stores. Future studies should conduct an in-depth analysis of privacy and security measures in mobile apps for older adults (eg, analyzing if they transmit data using services provided by Facebook or Google) [63].

### Scientific and Practical Implications

Since the user star rating is invalid to assess mobile app quality, publicly available expert mobile app ratings could help older adults as well as their relatives, caregivers, and health care professionals (eg, physicians) to select a high-quality mobile app. Publicly available MARS ratings by experts on a wide range of health topics on databases like Psyberguide and mHAD [71] could assist in informed health care decisions.

In the future, efficacy and effectiveness studies should be implemented for mobile apps. At present, there is a lack of high-quality studies that prove the long-term benefit, effectiveness, and safety of mobile app use for older adults [64,74]. In connection with efficacy and effectiveness studies, it could also be investigated which functions and properties of mobile apps have a particularly positive and long-term effect on the use of mobile apps by older adults. Based on this data, new evidence-based and effective mobile apps could be developed. Also, mobile apps whose effectiveness could be proven could be translated into other languages. Moreover, older adults should be involved as part of participative research in developing a new mobile app [91]. Taking end users into account increases the usability, uptake, and effectiveness of interventions [92]. After developing a new mobile app, it is essential to invest time in training tools, in-person training, user manuals, and support hotlines regarding the use of mobile app, as many older adults want to receive technical and social support for the installation, exploration, and learning of a mobile app [16,74,93-95]. Only making mobile apps available in the app stores will fail to optimize their use by older adults [96].

Promotion measures as reimbursement of costs of mobile apps with proven effectiveness through health care providers and targeted information campaigns on existing high-quality mobile apps for older adults and their relatives could help them to integrate high-quality mobile apps into their daily lives [93,97].

### Conclusion

The potential inherent in mobile apps to support a healthy, active, and safe life for older adults has not yet been sufficiently explored. The study was able to indicate that currently available mobile apps for older adults are on average of moderate overall quality. In particular, deficiencies could be found in information quality, evidence-based approach, data protection, and security measures. However, some mobile apps were of high quality, were based on evidence, and had sufficient data protection, and therefore, could provide suitable support. The user star rating and the obligation to pay fees did not provide valid orientation aids. Annually conducted reviews and publicly available expert mobile app ratings could help older adults and their relatives as well as caregivers to select a high-quality mobile app.

## Appendix

Multimedia Appendix 1 Preferred Reporting Items for Systematic Reviews and Meta-analyses 2009 checklist.

Multimedia Appendix 2 Included mobile apps with name, store, developer, version, price, user star rating, Mobile Application Rating Scale, German version (MARS-G), subdimensions and overall rating sorted by MARS-G overall rating.

## Abbreviations

ICC: intraclass correlation
MARS-G: Mobile Application Rating Scale, German version
PRISMA: Preferred Reporting Items for Systematic Reviews and Meta-analyses
