# Supplementary material for: Mobile Apps for Older Adults: Systematic Search and Evaluation Within Online Stores
Source: JMIR Aging. 2021 Feb 19;4(1):e23313. doi: 10.2196/23313 (PMC8081158; doi:10.2196/23313)
Supplement: Multimedia Appendix 2 [file aging_v4i1e23313_app2.pdf]

Multimedia Appendix

Table 1. Included mobile apps with name, store, developer, version, price, user star rating, Mobile Application Rating Scale, German version (MARS-G), subdimensions and overall rating sorted by MARS-G overall rating.

App name	App store	App developer	Version	App price in Euro	User star rating	MARS-G Engagement	Functionality	Aesthetics	Information	Overall rating
NeuroNation - Gehirnjogging	Google Play Store	NeuroNation	3.1.62	0.00	4.50	4.80	4.88	5.00	4.29	4.74
Lumosity	Google Play Store	Lumos Laps Inc.	22.04	0.00	4.20	4.60	4.88	4.83	4.21	4.63
DST - Demenz Test	App Store	Sebastian Dr. Horn	1.3	5.49	5.00	4.20	5.00	5.00	4.00	4.55
MindMate	App Store	MindMate LTD	5.5.1	0.00	5.00	4.70	4.50	4.83	4.07	4.53
Arznei & Medikamente Alarm	App Store	MediSafe Inc.	6.2.10	0.00	4.50	4.20	4.63	4.17	4.21	4.30
Constant Therapy	Google Play Store	Constant Therapy Inc.	4.9.4	0.00	4.20	4.40	4.38	4.00	4.00	4.19
Memory Helper Carer Assistance Tool	Google Play Store	Memory Helper Carer Assistance Tool	1.0.4	0.00	4.50	4.50	4.50	4.00	3.64	4.16
Ada - Deine Gesundheitshelferin	Google Play Store	Ada Health	2.39.0	0.00	4.70	4.40	4.63	4.17	3.43	4.16
Auguste	Google Play Store	Vollkorn Games	2.1.5	0.00	4.50	4.50	4.63	4.83	2.50	4.11

App name	App store	App developer	Version	App price in Euro	User star rating	MARS-G Engagement	Functionality	Aesthetics	Information	Overall rating
Mathe Senioren	App Store	Nicolas Lehovetzki	1.1	2.29	4.00	4.20	5.00	5.00	2.00	4.05
Peak - Gehirntraining	Google Play Store	Peaklabs	3.26.42	0.00	4.40	4.40	4.38	4.00	3.21	4.00
Senior Safety App, GPS Tracker – Fall Alerts & More	Google Play Store	97 Technologies	9.7	0.00	4.00	4.60	4.25	4.00	3.00	3.96
Brain Monitor: Letters	App Store	PeqApps	1.6	0.00	0.00	3.70	4.50	4.50	2.93	3.91
Familie Suchen - GPS Tracker	Google Play Store	Life360	18.6.0	0.00	4.50	3.80	4.00	4.00	3.71	3.88
Dementia - Cuomo	App Store	Cuomo	1.02	0.00	0.00	3.50	4.38	4.50	2.93	3.83
Dementia Risk Tool	Google Play Store	SICS Swedish ICT	1.21	0.00	4.90	2.80	4.75	4.33	3.21	3.77
Spiele für Senioren - Trainieren Sie Ihren Geist	App Store	Lisbon Labs	1.2	2.29	2.00	4.00	4.50	5.00	1.57	3.77

App name	App store	App developer	Version	App price in Euro	User star rating	MARS-G Engagement	Functionality	Aesthetics	Information	Overall rating
Trainiere dein Gehirn - Gedächtnisspiele	Google Play Store	Silver Senior Games	2.1.3	0.00	4.50	4.00	4.13	4.67	2.21	3.75
Trainiere dein Gehirn - Aufmerksamkeits-spiele	Google Play Store	Senior Games	1.2.3	0.00	4.50	3.80	4.25	4.67	2.14	3.71
Brain Monitor: Scramble	App Store	PeqApps	1.6	0.00	0.00	4.00	3.88	4.33	2.57	3.69
Alzheimer's Life Therapy	App Store	Alzheimer's Light	1.3.2	0.00	5.00	4.10	4.13	3.67	2.86	3.69
SymptomGuide TM Dementia	App Store	DGI Clinical	0.9.0.0	0.00	0.00	3.40	4.25	4.00	3.00	3.66
MyTherapy Tabletten Erinnerung	App Store	smartpatient GmbH	3.34	0.00	4.80	3.90	4.38	4.00	2.29	3.64
Emergency SOS Safety Alert - Personal Alarm App	Google Play Store	appCDM Health & Fitness	0.25	0.00	3.40	3.00	4.38	4.17	2.86	3.60

App name	App store	App developer	Version	App price in Euro	User star rating	MARS-G Engagement	Functionality	Aesthetics	Information	Overall rating
Oscar Senior	App Store	Oscar Senior s.r.o.	3.3.0	0.00	3.60	4.20	4.75	4.17	1.21	3.58
Dementia Guide Expert	App Store	University of Illinois	1.1.2	0.00	0.00	3.00	4.50	3.33	3.43	3.57
Memory box	Google Play Store	SCI AB	1.7	0.00	5.00	3.50	3.63	4.00	3.00	3.53
Vergrößerungsglas - Lupe app	Google Play Store	Aexol	1.6	5.49	4.00	3.80	4.75	4.67	0.86	3.52
Life Begins At ... The Retiree	App Store	Magzter Inc.	6.16	0.00	0.00	3.10	4.00	4.50	2.43	3.51
Trainiere dein Gehirn - visuell-räumliche Spiele	App Store	Senior Games	1.1.5	0.00	4.50	4.00	4.25	3.83	1.79	3.47
Activities for care: elderly dementia LD seniors	App Store	RemindMecare Medical	1.0.0	0.00	4.90	3.40	3.25	4.67	2.50	3.45
iHELP Personal & Family Safety	App Store	iHELP	3.5.9	0.00	4.50	3.60	4.13	4.17	1.86	3.44

App name	App store	App developer	Version	App price in Euro	User star rating	MARS-G Engagement	Functionality	Aesthetics	Information	Overall rating
Phantasiereisen für Senioren	Google Play Store	start2dream.de	1.5	0.00	4.80	3.30	4.75	3.33	2.29	3.42
EasyTalkerFree	App Store	Janus Anderson	1.0	0.00	0.00	2.90	3.75	4.33	2.64	3.41
Apotheke vor Ort	App Store	Wort & Bild Verlag Konradshöhe GmbH & Co. KG	8.2.5	0.00	2.60	3.60	3.63	3.50	2.86	3.40
Dementia Personal Outcomes	Google Play Store	Scottish Social Services Council Education	2.0	0.00	0.00	2.50	3.38	4.00	3.64	3.38
Young Onset Dementia (YOD)	App Store	Leicestershire Partnership NHS Trust with LHIS	1.0	0.00	5.00	2.70	3.25	3.83	3.71	3.37
Dementia 101 - Memory loss and Family Care Guide	App Store	Xi Zhang	1.0	3.49	5.00	2.60	4.75	3.67	2.43	3.36

App name	App store	App developer	Version	App price in Euro	User star rating	MARS-G Engagement	Functionality	Aesthetics	Information	Overall rating
Senior Fitness – Home workout for old and elderly	Google Play Store	K2 Labs Health & Fitness	1.1.1	0.00	4.20	3.20	3.38	3.67	3.14	3.35
Senior Next	App Store	Online Connections Inc.	1.0.23	0.00	4.00	4.10	4.38	3.67	1.21	3.34
Spiele für Gedächtnis	Google Play Store	Maple Media	3.6.30	0.00	4.50	3.70	4.00	3.67	1.86	3.31
Dementia Solutions	Google Play Store	Sunnsoft Medical	0.5	0.00	5.00	3.50	3.50	3.50	2.71	3.30
Wortsuche	Google Play Store	Senior Games	1.1.8	0.00	4.60	3.90	4.38	4.00	0.93	3.30
HandHelp - Life Care App/ barrierefreier Notruf	Google Play Store	App-Sec-Network UG	2.0.1	0.00	4.30	3.40	3.50	4.00	2.00	3.23
Dementia Screener	Google Play Store	Bioinformatics Research Group BIRG Health & Fitness	2.1	0.00	2.70	2.80	4.25	3.17	2.64	3.21

App name	App store	App developer	Version	App price in Euro	User star rating	MARS-G Engagement	Functionality	Aesthetics	Information	Overall rating
Rimentia - Gehirntraining	Google Play Store	AFASH Apps	1.2	0.00	4.00	3.60	4.00	4.17	0.93	3.17
Aphasie, Schlaganfall und Demenz Therapie	Google Play Store	ImagiRation LLC	5.5.3	0.00	4.20	3.70	3.00	3.33	2.43	3.12
Medication | Pill Reminder App	Google Play Store	Nextscrum Apps	1.0.19	0.00	4.50	3.70	3.13	3.50	2.00	3.08
Senior Safety Phone – Big Icons Launcher	Google Play Store	Deskshare Inc.	2.8	0.00	3.90	3.60	4.63	3.33	0.71	3.07
Dementia Info	Google Play Store	Programming Is Fun	1.0	0.00	0.00	2.70	3.88	3.17	2.36	3.02
AlerteChute	Google Play Store	Jginformatique	1.0.12	18.99	4.00	3.60	4.00	3.33	1.14	3.02
Care and Connect: Dementia Friendly Places	Google Play Store	University of Newcastle upon Tyne	1.2	0.00	3.80	3.10	4.00	3.67	1.29	3.01
SitFit Exercise	App Store	Candlhat Studios	1.1	0.00	2.00	2.60	3.50	3.33	2.57	3.00

App name	App store	App developer	Version	App price in Euro	User star rating	MARS-G Engagement	Functionality	Aesthetics	Information	Overall rating
9zest Parkinson-Therapie	App Store	9zest Inc.	3.2.0	0.00	4.80	3.40	3.25	3.33	1.71	2.92
Are you okay today	App Store	HANBUNCO	2.2	0.00	4.10	2.80	4.75	3.17	0.79	2.88
Senioren Video Überwacher	App Store	Master App Solutions	2.1	6.99	4.40	2.40	3.75	4.00	1.29	2.86
Senior einfaches Telefon	Google Play Store	Adcoms	2.0	0.00	3.70	2.00	4.38	3.67	1.36	2.85
Dementia Diary	App Store	Mark Thomas	1.3	1.09	4.00	2.40	4.38	3.33	1.29	2.85
Alzheimer's Speed of Processing Game - ASPEN	Google Play Store	Tiny Happy Steps	1.7.0	0.00	3.90	2.20	4.75	3.50	0.93	2.84
Senior Discounts + Coupons Free	Google Play Store	Big Books Apps. LLC	1.8	0.00	4.20	3.60	3.88	1.67	1.79	2.73

App name	App store	App developer	Version	App price in Euro	User star rating	MARS-G Engagement	Functionality	Aesthetics	Information	Overall rating
Daily Senior Fitness Exercise	Google Play Store	EBMACS Health & Fitness	1.2	0.00	3.60	2.40	4.00	3.17	1.36	2.73
Senior Homescreen	Google Play Store	Endran Personalisation	1.2.1	0.00	3.80	2.60	4.00	3.33	0.86	2.70
Dementia & Alzheimer's Memory Diagnosis Test: MMSE	Google Play Store	Dementia Analysis Health & Fitness	1.0	0.00	3.60	2.00	4.25	3.50	0.93	2.67
Senior Assist Free	App Store	RedFlag Technologies	1.0	0.00	4.00	3.10	3.75	2.50	1.29	2.66
Riesige Tastatur	Google Play Store	Apps Technologies	5.0	0.00	4.30	2.20	4.13	3.33	0.64	2.58
Visual Hearing Aid	App Store	Berry Wing LLC	1.0.4	0.00	4.10	2.80	3.88	2.83	0.79	2.57
Senior Chess	Google Play Store	Leen Ammeraal Board	2.02	0.00	4.10	3.20	3.75	2.00	1.29	2.56
Memory Game Brain for dementia	Google Play Store	Team Jin	04.17	0.00	4.60	3.10	3.88	2.33	0.86	2.54

App name	App store	App developer	Version	App price in Euro	User star rating	MARS-G Engagement	Functionality	Aesthetics	Information	Overall rating
Sudoku: Senior Puzzle	App Store	zhong chen	1.2	0.00	4.00	2.90	3.88	2.67	0.64	2.52
Tastatur für Senioren	Google Play Store	ctpg567	2.2	0.00	4.00	2.50	3.63	3.17	0.79	2.52
Exercise Tips For The Elderly	App Store	TrainTech USA. LLC	1.0	3.49	0.00	1.90	3.63	2.50	1.86	2.47
Senior Menu	Google Play Store	Willem Me	1.0	0.00	5.00	2.80	3.38	2.83	0.86	2.47
GROßE TASTEN SENIOREN TELEFON	Google Play Store	Anna G.	1.37	0.00	3.20	2.50	3.50	3.17	0.64	2.45
Gedächtnisübung für Alzheimer	Google Play Store	Büyük Beyaz Hamsi	4.1.0	0.00	4.40	2.40	3.88	2.50	0.93	2.43
Accessible Alzheimers and Dementia Care	App Store	AHCGLOBAL	1.0	0.00	0.00	1.70	3.38	2.83	1.79	2.42
Locate Me – App for children and elderly citizen	Google Play Store	Perfect Fact Maps & Navigation	1.0.0	0.00	4.80	1.70	3.50	3.17	1.14	2.38

App name	App store	App developer	Version	App price in Euro	User star rating	MARS-G Engagement	Functionality	Aesthetics	Information	Overall rating
Senioren. Rentner. Demenz. Uhr. App. Kalender	Google Play Store	Aruso	1.0	12.99	3.00	2.80	3.13	2.83	0.71	2.37
Senior Safety Phone	App Store	Contacts by Company. Inc.	1.1	0.00	3.80	2.70	2.88	3.17	0.71	2.36
Gabble	Google Play Store	Denis E.	1.0.2	0.00	0.00	3.00	3.25	2.33	0.43	2.25
Memory Games for Elderly	Google Play Store	Nice.Jirayu2554	1.0	0.00	2.00	2.20	3.38	2.33	0.36	2.07
Dementia/Digital Diary/Clock	Google Play Store	Fashmel Health & Fitness	4.2	0.00	4.40	2.60	3.13	1.33	0.64	1.93
Dementia Player Demo	Google Play Store	Stephen C Adams Music & Audio	11.0	0.00	0.00	1.90	3.63	1.00	0.93	1.86
Dementia_Counter	Google Play Store	Stephen C Adams	2.0	0.00	0.00	1.00	1.63	2.33	0.71	1.42
